# Initial Treatment of IDH-Wildtype Glioblastoma in Adults Older Than 70 Years

**DOI:** 10.7759/cureus.47602

**Published:** 2023-10-24

**Authors:** Jing Bao, Zhenjiang Pan, Shepeng Wei

**Affiliations:** 1 Neurosurgery, Shidong Hospital of Yangpu District, Shanghai, CHN

**Keywords:** immunotherapy, alternating electric fields, temozolomide, mgmt, surgical resection, idh-wildtype glioblastoma

## Abstract

The incidence of glioblastoma, the most common malignant primary brain tumour in adults, increases after the age of 40 and peaks in adults aged 75-84 years. Initial management involves maximising surgical resection while preserving neurologic function. *IDH* mutations and MGMT promoter methylation should be checked in tumour samples. Radiation and temozolomide constitute initial treatment for newly diagnosed glioblastoma patients with good functional status. It is suggested that patients who have received concurrent and adjuvant temozolomide treatment should undergo six cycles of adjuvant monthly temozolomide, as opposed to a more extended treatment regimen. Low-intensity alternating electric field therapy improved survival in a large randomised trial. We provide a detailed review, providing the latest treatment viewpoint for *IDH*-wildtype glioblastoma and including the current situation of immunotherapy. The treatment ideas and methods reviewed here would be of help to physicians when they encounter patients with this kind of *IDH*-wildtype glioblastoma in clinical practice.

## Introduction and background

The incidence of glioblastoma, the most common malignant primary brain tumour in adults, increases after 40 years of age and peaks in adults aged 75-84 years [1,2]. In adults, the classification of diffuse gliomas is determined by the World Health Organization (WHO) system, which considers both histological and molecular features. According to the 2021 classification by the WHO, glioblastomas are currently characterized as *IDH*-wildtype. In previous literature, the majority of these tumours were referred to as “primary glioblastomas” [3,4]. In contrast, *IDH*-mutant astrocytomas that progress to WHO grade 4 lesions are neoplasms that would have previously been referred to as “secondary glioblastoma.”

It must be emphasized again that the existing classification system established by the WHO limits the identification of glioblastoma to the most severe grade of diffuse gliomas that lack *IDH* mutations [3,4]. In this review, we discuss the treatment of *IDH*-wildtype glioblastoma only in adults aged more than 70 years.

Here, we provide a detailed review of the latest treatment viewpoint for *IDH*-wildtype glioblastoma and the current status of immunotherapy. These treatment ideas and methods should be considered when encountering a patient with this type of *IDH*-wildtype glioblastoma.

## Review

*IDH *gene

In 2008, the discovery of somatic mutations that affect the active site of the Krebs cycle enzyme, IDH1, was initially observed during a comprehensive genomic analysis of tumor samples from human glioblastoma. Among the 149 tumors analyzed, 18 exhibited modifications in the *IDH1* gene, predominantly at the R132 residue [5].

The findings mentioned have been corroborated by subsequent studies, which have also discovered less common mutations in *IDH1* and the related gene,* IDH2*, in glioma. Furthermore, these studies have expanded the range of affected tumors to encompass a significantly larger proportion of lower-grade gliomas compared to glioblastoma [6]. The occurrence of these mutations represents the earliest documented event in diffuse gliomagenesis [5,7].

Formation of *IDH*-wildtype glioblastoma

High-grade gliomas are believed to originate from neural progenitor cells; however, the specific differentiation stage of these target cells, i.e., whether they are stem cells or progenitor cells, remains uncertain. In glioblastoma characterized by wild-type *IDH*, investigations involving mouse models, molecular genetic analyses of tumor tissue from patients, and biopsies from the adjacent subventricular zone (SVZ) and normal tissue, postulates astrocyte-like neural stem cells in the SVZ, harboring somatic driver mutations at a low level, as the cellular source [8]. Over time, these cells can migrate and accumulate further somatic mutations, ultimately resulting in the formation of *IDH*-wildtype glioblastoma in remote areas of the brain.

High-grade gliomas encompass multipotent tumor stem cells, which play a pivotal role in the colonization and subsequent repopulation of these tumors [9-12]. The presence of these tumor stem cells holds potential therapeutic implications, as treatments that fail to eliminate the tumor stem cells will prove ineffective in eradicating the tumor.

Glioblastoma is the prevailing malignant primary brain tumor in the adult population, typically manifesting in 55-60 years of age. According to the 2021 update of the WHO Classification of Central Nervous System (CNS) Tumors, the categorization of tumors previously referred to as glioblastoma was modified to include two diagnoses based on the presence or absence of *IDH* mutation [4]: (i) glioblastoma, *IDH*-wildtype, CNS WHO grade 4 and (ii) astrocytoma, *IDH*-mutant, CNS WHO grade 4.

Treatment of *IDH*-induced wildtype glioblastoma

The majority of individuals diagnosed with glioblastoma receive a combined-modality treatment strategy that involves the administration of adjuvant postoperative radiation therapy (RT) and adjuvant chemotherapy after the initial surgical procedure. Despite receiving intensive treatment, glioblastoma exhibits a substantial propensity for recurrence and is associated with a generally unfavorable prognosis, typically resulting in a median overall survival of 1.5-2 years.

Surgical Resection

The preferred surgical approach for patients diagnosed with glioblastoma involves achieving maximal surgical resection while ensuring the preservation of neurologic function. The utilization of contemporary surgical methodologies, such as neuro-navigation, intraoperative MRI suite, and the application of aminolevulinic acid (ALA) dye for tumor delineation during surgical procedures, should be implemented in medical facilities equipped with these advanced instruments. While it is generally preferable to perform a gross total resection, there may be instances where a subtotal resection or biopsy alone is necessary, depending on the tumor’s location and size.

Adjuvant RT

Adjuvant RT is a customary element for treating glioblastoma and has been demonstrated to enhance local control and overall survival following surgical removal.

RT is administered to the tumor along with a surrounding area of radiographically normal tissue, which is intended to include infiltrating tumor cells. Intensity-modulated RT (IMRT), which includes the specific subtype known as volumetric modulation arc therapy (VMAT), is becoming increasingly popular as the standard technique for delivering RT. VMAT allows for optimal coverage of the intended target area(s) with minimal exposure of surrounding healthy tissues to high doses of radiation. IMRT and VMAT enhance the radiation dose distribution to tumor regions in close proximity to radiation-sensitive structures, such as the optic chiasm and optic nerves. This is particularly important because radiation dose constraints necessitate a compromise between minimizing the dose to nearby tumor tissue and adhering to dose limits for these sensitive structures.

The standard RT dosages administered for glioblastoma typically comprise 60 Gy delivered in 2 Gy fractions, with limited evidence to suggest that increasing the dose beyond 60 Gy yields additional advantages [13-15]. 

The utilization of proton beam RT is increasingly prevalent and has emerged as a standard treatment modality for pediatric brain tumors, specifically medulloblastoma. There exists a scarcity of data regarding the potential efficacy of high-grade gliomas, particularly in relation to conformal photon RT, let alone any indication of superiority. While the utilization of protons for radiation delivery allows for precise targeting, it may be more appropriate to focus on minimizing side effects related to RT when considering its potential application in treating glioblastoma. The ongoing multicenter trial (NCT02179086) is currently investigating the role of dose-escalated protons.

Adjuvant Systemic Therapy

In the case of glioblastoma, it is recommended to conduct tests on tumor samples to determine the presence of O6-methylguanine-DNA methyltransferase (MGMT) promoter methylation and *IDH* type 1 or type 2 mutations, particularly in patients younger than 55 years

MGMT-methylated tumors: A European Organisation for Research and Treatment of Cancer (EORTC)/National Cancer Institute of Canada (NCIC) open-label trial was the first to show that concurrent and adjuvant treatment with temozolomide was effective in adults with glioblastoma. In this trial, 573 patients aged 18-70 years were randomly assigned to receive either involved-field RT alone or radiation plus concurrent daily temozolomide, followed by up to six monthly cycles of adjuvant temozolomide [16]. With a median follow-up period exceeding five years, the incorporation of temozolomide alongside RT demonstrated a notable enhancement in the median overall survival when compared to RT alone. The median overall survival was 14.6 months with the addition of temozolomide, compared to 12.1 months with RT alone. This improvement in survival was quantified by a hazard ratio (HR) of 0.63 (95%CI 0.53-0.75) [16]. Long-term follow-up showed that survival was still better in the temozolomide group at two years (27% vs. 11%) and five years (10% vs. 2%) [17]. The efficacy of adjuvant temozolomide was observed across all patient subsets, including individuals aged ≥ 60 years and those with other unfavorable prognostic factors [16-19]. Comparable outcomes were observed in a subsequent, more limited phase II clinical study involving patients diagnosed with glioblastoma [19].

In the CeTeG/NOA-09 trial, 650 patients were initially screened, among whom, 141 patients aged 18-70 years with MGMT-methylated glioblastoma were selected for enrollment and randomly assigned to one of two treatment groups. The first group received a combined regimen of lomustine and temozolomide, which was administered during and after RT. This regimen consisted of up to six cycles of lomustine at a dosage of 100 mg/m^2^ on day 1 and temozolomide at a dosage of 100 mg/m^2^ on days two to six. The second group received standard therapy, which involved daily temozolomide treatment alongside RT, followed by up to six cycles of adjuvant monthly temozolomide [20]. Within the modified intention-to-treat (mITT) population, the comparison between lomustine/temozolomide and standard temozolomide revealed comparable median overall survival rates (37.9 months vs. 31.4 months, HR: 0.90, 95%CI: 0.58 to 1.41). Using inverse probability weights, an adjusted mITT analysis on these 129 patients revealed a nonsignificant trend toward improved survival in the lomustine/temozolomide arm (HR: 0.74, 95%CI: 0.47-1.17) and no significant difference in progression-free survival (HR: 0.99, 95%CI 0.47-1.44)

The findings of these studies are thought-provoking and provide evidence to suggest that the combination therapy of lomustine and temozolomide may enhance survival rates when compared to standard temozolomide treatment in a specific subgroup of younger, healthy patients with MGMT-methylated glioblastoma. Nevertheless, the use of combination therapy has been linked to increased incidences of nausea and hematologic toxicity, which may result in patients and healthcare professionals opting for the conventional temozolomide treatment approach until additional research is conducted.

It is recommended that patients who have recently been diagnosed with MGMT-methylated glioblastoma and are ≤ 70 years old be treated with concurrent temozolomide along with RT, followed by monthly adjuvant temozolomide. In younger, physically healthy patients with MGMT-methylated tumors, a combination of temozolomide and lomustine, along with RT, can be considered as an alternative treatment option. However, the available data on its effectiveness are inconclusive, and there is a possibility of increased toxicity.

MGMT-unmethylated tumors: Patients with tumors that are unmethylated in the *MGMT* gene exhibit a bleak prognosis and limited response to conventional treatment methods. Therefore, it is strongly recommended that these patients consider enrolling in clinical trials. In the absence of a clinical trial setting, we propose the administration of temozolomide in conjunction with RT for most patients diagnosed with MGMT-unmethylated glioblastoma. This recommendation is derived from the findings of the EORTC/NCIC trial, in which prospective knowledge of MGMT status was unavailable [16,17].

A retrospective analysis was conducted on 206 patients who had participated in the EORTC/NCIC trial to determine their MGMT status. The results showed that in the 114 patients with MGMT-unmethylated tumors, the inclusion of temozolomide in RT resulted in a slight difference in survival rates. However, this difference was not statistically significant, as evidenced by the two-year survival rates of 15% vs. 2%, respectively, and the median overall survival of 12.7 vs. 11.8 months, respectively (HR: 0.69, 95%CI: 0.47-1.02) [21].

A previous study presented the findings of a phase II clinical trial involving 182 patients who were diagnosed with newly acquired glioblastoma and exhibited unmethylated MGMT. The trial aimed to compare the efficacy of two treatment regimens: one involving the administration of bevacizumab during RT followed by bevacizumab along with irinotecan and the other involving RT alongside concurrent and adjuvant temozolomide. This study was randomized and unblinded in nature [22].

A distinct clinical study was conducted to compare the efficacy of RT combined with nivolumab with that of RT combined with concurrent and adjuvant temozolomide. The results of this trial indicated that the nivolumab arm exhibited a lower survival rate than the temozolomide arm (median overall survival: 13.4 vs. 14.9 months, respectively; HR: 1.31, 95%CI: 1.09-1.58) [23].

MGMT status unknown: The determination of MGMT methylation status through assays is challenging in a significant proportion of patients, which is primarily attributed to inadequate tissue availability. This issue is particularly prominent among individuals who have undergone stereotactic biopsy procedures. It is recommended to administer temozolomide in conjunction with RT in cases where the status of MGMT is uncertain during postoperative decision-making and when the patient is otherwise eligible for standard therapy. The reasoning is based on the fact that temozolomide is not only safe and easy to take, but is also expected to improve survival in a clinically important way for 30-40% of patients who are expected to have an MGMT-methylated tumor, for which there are no better treatment options [17].

Limited Function of Bevacizumab

Bevacizumab is a monoclonal antibody that binds to vascular endothelial growth factor (VEGF). Bevacizumab is not recommended for routine use in patients with newly diagnosed glioblastoma [24]. Although bevacizumab exhibits strong antiedema properties, which can enhance clinical function and decrease the need for glucocorticoids in specific patients, its use as an initial therapy does not enhance overall survival and instead raises the likelihood of experiencing adverse effects [25].

Early administration of bevacizumab is considered a supportive pharmacological intervention for a specific group of patients with large, non-resectable tumors. The AVAglio study involved the random assignment of 921 patients to two groups: one receiving bevacizumab and the other receiving a placebo. Both groups received RT and temozolomide treatments [26]. The administration of bevacizumab resulted in a statistically significant increase in median progression-free survival in patients compared to those who received a placebo (10.6 months vs. 6.2 months, HR: 0.64, 95%CI: 0.55-0.74).

A study titled “RTOG 0825” involved the random allocation of 637 patients to two groups: one receiving bevacizumab and the other receiving a placebo. This treatment was administered starting with the fourth week of standard chemoradiation with temozolomide. Following this, the patients underwent 6-12 cycles of maintenance temozolomide along with either bevacizumab or placebo [27]. The administration of bevacizumab resulted in a statistically significant improvement in median progression-free survival compared to the placebo group (10.7 months vs. 7.3 months, p = 0.007). However, it is important to note that this outcome did not reach the predetermined level of significance, which was set at p < 0.004. Additionally, there was no significant difference observed in the median overall survival between patients who received bevacizumab and those who received a placebo (15.7 months vs. 16.1 months, p = 0.21). An association was observed between MGMT promoter methylation and progression-free survival (14 months vs. eight months) and overall survival (23 months vs. 14 months), irrespective of the treatment administered. Patients who received bevacizumab treatment experienced a higher incidence of severe adverse events, notably neutropenia, hypertension, and thromboembolism, and exhibited a greater symptom burden, a lower quality of life, and a more frequent deterioration in neurocognitive function than the group on placebo [28].

Administration of Systemic Therapy

Temozolomide: During radiation, 75 mg/m^2^ of temozolomide is administered every day, seven days a week. Temozolomide should not be administered if the number of platelets falls below 100,000/μL or the number of absolute neutrophils (ANC) falls below 1500/μL, as these numbers can drop quickly.

The first cycle of temozolomide after RT usually starts four weeks after the last treatment and is given at a dose of 150 mg/m^2^ every day for five days out of a 28-day cycle. If the blood counts are good, the dose for cycles 2-6 would be 200 mg/m^2^. In the adjuvant setting, other schedules of temozolomide have not been found to work better than the standard schedule of five days every 28 days [29-31].

Typically, the standard approach involves administering a maximum of six cycles of post-radiation temozolomide, following the methodology outlined in the original study that established temozolomide as the accepted standard treatment [16]. The rationale for this approach is supported by empirical data obtained from a phase II randomized trial. In this trial, 159 patients diagnosed with glioblastoma who had not experienced disease progression following six cycles of adjuvant temozolomide were randomly allocated into two groups. The first group served as the control and ceased temozolomide treatment, whereas the second group continued receiving temozolomide for a maximum of 12 cycles [32]. With a median follow-up duration of 33 months, the groups exhibited similar progression-free survival, and, in fact, the extended temozolomide group demonstrated a non-significant trend toward worse overall survival (HR: 1.3, 95% CI:] 0.90-1.88). Secondary analyses found no correlation between MGMT methylation status or disease severity and benefit from additional temozolomide cycles, despite limited power to eliminate clinically significant differences.

A retrospective study of 624 patients in four randomized trials found that receiving more than six cycles of adjuvant temozolomide improved progression-free survival, particularly in MGMT-methylated tumors (HR: 0.65, 95%CI: 0.50-0.85), but did not affect overall survival (HR: 0.92, 95%CI: 0.71-1.19), even in the subgroup (HR: 0.89, 95%CI: 0.63-1.26) [33].

Temozolomide and lomustine: In the CeTeG/NOA-09 trial, temozolomide and lomustine were administered in six-week cycles starting in the first week of RT (lomustine (1-(2-chloroethyl)-3-cyclohexyl-1-chloroethylnitrosourea (CCNU)) 100 mg/m^2^ taken by mouth on day 1, and temozolomide 100 mg/m^2^ taken by mouth on days 2-6) [9]. Starting on day 21, complete blood counts should be performed every week in the next cycle. A basic metabolic panel and biochemical tests of the liver should be performed at the beginning and middle of each cycle.

In the study by Weller et al., they found a non-significant increase in the incidence of grade ≥ 3 hematologic toxicity when temozolomide was combined with lomustine compared to temozolomide alone. The rates of hematologic toxicity were 36% and 29%, respectively [34]. Toxicity was predominantly observed in patients undergoing concurrent chemoradiotherapy. Patients who subsequently received one or more adjuvant cycles of chemotherapy had a 42% likelihood of experiencing recurrent hematologic toxicity. Being female (odds ratio of 2.5) and advancing age were identified as two risk factors associated with hematologic toxicity.

Alternating Electric Fields

In 2011, a portable medical device capable of generating tumor-treating fields (TTFields) was introduced for the treatment of recurrent glioblastoma [35]. A subsequent open-label randomized trial indicated that the device, when used in conjunction with monthly temozolomide, enhanced both progression-free and overall survival in patients with newly diagnosed glioblastoma in the post-radiation stage [36,37].

The evidence supporting the use of TTFields in the initial treatment of glioblastoma was derived from an unblinded, multicenter international trial, in which 695 patients with newly diagnosed glioblastoma were randomly assigned to receive either monthly temozolomide plus TTFields or monthly temozolomide alone. This random assignment was performed at a 2:1 ratio [36]. The treatment was administered after the completion of standard RT and concurrent daily temozolomide. For inclusion, patients were required to have completed concurrent radiation and daily temozolomide treatment without progression and be enrolled within seven weeks. The main endpoint was progression-free survival. During an interim analysis of the first 315 patients with ≥ 18 months of follow-up, the trial was halted early for benefit. At this point, enrollment had reached the intended 695 patients.

In another study by Stupp et al., the baseline patient characteristics were similar in the test group (n = 466) and control (n = 229) arms [37]. The median age of the study participants was 56 years, the Karnofsky performance status (KPS) was 90, and 54% underwent gross total resection. MGMT promoter methylation status was found in 82% of patients and methylated in 41%. Magnetic resonance imaging (MRI) images were centrally reviewed by two blinded radiologists using the Macdonald criteria. Both groups had a median diagnosis-to-randomization time of 3.8 months. At a median follow-up of 40 months, patients assigned to the TTFields device had better progression-free survival than those assigned to temozolomide alone (6.7 vs. 4.0 months, HR: 0.63, 95% CI: 0.52-0.76). The overall survival from randomization also improved in the patients assigned to the TTFields device (20.9 vs. 16.0 months, HR: 0.63, 95% CI: 0.53-0.76).

The results of a secondary analysis examining quality of life outcomes revealed no discernible distinction between the groups at the 9- and 12-month time points [38, 39].

The biological activity of the therapy is purported to arise from an antimitotic effect induced by the alternating electric fields, which apply forces to charged tubulin subunits, thereby impeding the assembly of the mitotic spindle [40,41].

Follow-up and monitoring

Assessment of Response and Progression

It is necessary to evaluate both the initial response to treatment and the subsequent evidence of disease progression to make informed patient management decisions. To evaluate disease progression, brain MRI with contrast is commonly performed approximately one month after the conclusion of RT, followed by subsequent evaluations every two months during adjuvant temozolomide treatment.

The Macdonald criteria, which rely on measuring areas of contrast enhancement, have long been used for the formal response assessment of high-grade gliomas [42]. The Response Assessment in Neuro-Oncology (RANO) working group has proposed new criteria to deal with difficulties in assessing patients with pseudoprogression or progressive disease in patients with non-enhancing lesions [43].

Prognosis

Age, KPS, MGMT status, and various molecular genetic alterations are the primary prognostic factors that significantly influence the outcome of patients diagnosed with glioblastoma. As mentioned previously, the prognosis is also influenced by the extent of initial surgical resection.

The median overall survival of patients diagnosed with glioblastoma in population-based studies is estimated at 10-12 months [44-46]. The one-year survival rates for glioblastoma cases diagnosed between 2002 and 2010 in the United States and Taiwan varied between 38% and 50%, while the five-year survival rates ranged from 5% to 10% [47].

Gittleman et al. built an online nomogram derived from the clinical trial population and validated on another cohort [48]. To estimate the six-month, 12-month, and 24-month survival probability, the nomogram considered patient age at diagnosis, sex, KPS, extent of resection, and MGMT status.

The recursive partitioning analysis (RPA) classification of glioblastoma is an established prognostic tool that was developed and validated before the introduction of temozolomide. Validation was conducted on a cohort of patients who predominantly received RT without concurrent temozolomide treatment [49]. The classification estimates median survival in three subgroups: RPA class III (age < 50 years, KPS ≥ 90), 17.1 months (12-month survival 70%); RPA class IV (age <50, KPS <90), 11.2 months (12-month survival 46%); and RPA class V (all others), 7.5 months (12-month survival 28%).

The MGMT enzyme repairs DNA after alkylating agent chemotherapy. During tumor development, methylation of the *MGMT* gene promoter can prevent DNA repair and increase the efficacy of alkylating agent chemotherapy. A meta-analysis of 11 studies, conducted to investigate the prognostic significance of the MGMT promoter status, revealed that a methylated MGMT promoter was significantly associated with improved progression-free survival (HR: 0.56; 95%CI: 0.32-0.80) and overall survival (HR: 0.50, 95%CI: 0.35-0.66) [50].

The potential correlation between MGMT promoter methylation and chemotherapy efficacy in patients with glioblastoma has yet to be substantiated through a prospective investigation [21,51]. In a randomized trial, it was seen that patients with MGMT-methylated tumors had a two-year overall survival rate of 49% compared to 15% in those with unmethylated tumors [33]. Patients with a methylated promoter benefited more from the addition of temozolomide to radiation than those with an unmethylated promoter.

Immunotherapy

The initial use of checkpoint inhibitors, specifically pembrolizumab and nivolumab, in individuals with high-grade glioma has demonstrated limited efficacy [52-54], and the use of checkpoint inhibitors in a non-targeted population of individuals experiencing recurring high-grade glioma is not advised outside clinical trial settings. The assessment of programmed death ligand 1 (PD-L1) expression does not provide discriminatory value in predicting treatment response among patients [53,54].

The open-label CheckMate 143 trial, which involved 369 patients with glioblastoma experiencing their first recurrence, represents the most extensive investigation of single-agent nivolumab. These patients were randomly assigned to receive either nivolumab (3 mg/kg) or bevacizumab (10 mg/kg) every two weeks [55]. With a median follow-up duration of 9.5 months, the overall survival rates were comparable between the groups receiving nivolumab and bevacizumab (9.8 months vs. 10.0 months; HR: 1.04, 95%CI: 0.83-1.3). However, the group treated with bevacizumab exhibited a higher objective response rate (25% vs. 8%). A preliminary trial with randomization was conducted on patients undergoing resection of recurrent glioblastoma [56]. The findings of this trial indicated that initiating pembrolizumab treatment before surgery may lead to better outcomes than initiating therapy after surgery. However, additional research is required to further investigate this matter.

Exploration of combination therapy has also been undertaken. In a phase I clinical trial involving 40 patients with recurrent glioblastoma, the tolerability of nivolumab was found to be superior to that of combination therapy with ipilimumab [53]. In addition, a partial response was observed in three patients, with one patient responding positively to nivolumab monotherapy and two patients responding positively to combination therapy. Furthermore, an additional eight patients exhibited a state of stable disease for a minimum duration of 12 weeks, with two of them being treated solely with nivolumab. The presence of PD-L1 expression at ≥ 1% was observed in 68% of tumor specimens; however, no significant correlation was found between PD-L1 expression and clinical response. A subsequent phase I clinical trial demonstrated the viability of integrating pembrolizumab and bevacizumab with hypofractionated stereotactic reirradiation [57].

A favorable response to nivolumab in two children with recurrent glioblastoma associated with constitutional mismatch repair-deficiency syndrome has been documented in a case report [58]. In contrast, there is no apparent association between a hypermutation phenotype in recurrent glioblastoma and the response to checkpoint inhibitors, as observed in experimental studies and retrospective analyses of patient cohorts [59].

## Conclusions

Glioblastomas are classified into two types based on *IDH* mutation status: glioblastoma, *IDH*-wildtype, grade 4, and astrocytoma, *IDH*-mutant, grade 4. The incidence of glioblastoma, the most common malignant primary brain tumor in adults peaks in adults aged 75-84 years. Initial management involves maximizing surgical resection while preserving neurological function. *IDH* mutations and MGMT promoter methylation should be checked in tumor samples. Radiation and temozolomide constitute the initial treatment for patients with newly diagnosed glioblastoma with good functional status. In terms of *IDH*-wild-type glioblastoma in adults < 70 years of age, our recommendations are as follows: postoperative RT with concurrent and adjuvant temozolomide is recommended for MGMT-methylated glioblastoma and MGMT-unmethylated glioblastoma, and for patients with unknown MGMT methylation status. Radiation plus temozolomide is the standard of care due to the clinically significant survival improvement observed as well as its relative safety and tolerability and a lack of alternatives for unmethylated tumors. Patients who have received concurrent and adjuvant temozolomide treatment should undergo six cycles of adjuvant monthly temozolomide, as opposed to a more extended treatment regimen. Low-intensity alternating electric field therapy (TTFields) has been seen to have improved survival. Although interested patients are encouraged to use the device, carrying a device and shaving the scalp during treatment may be too much for some. Preliminary use of checkpoint inhibitors, namely pembrolizumab and nivolumab, has shown restricted effectiveness in patients diagnosed with high-grade glioma. Consequently, the administration of checkpoint inhibitors to a non-specific cohort of patients suffering from recurrent high-grade glioma is not recommended unless they are enrolled in a clinical trial.
